# An efficient identification strategy of clonal tea cultivars using long-core motif SSR markers

**DOI:** 10.1186/s40064-016-2835-8

**Published:** 2016-07-22

**Authors:** Rang Jian Wang, Xiang Feng Gao, Xiang Rui Kong, Jun Yang

**Affiliations:** Institute of Tea, Fu Jian Academy of Agricultural Sciences, 1 Hu Tou Yang Road, She Kou, Fu An, 355015 Fu Jian China; Fu Jian Branch, National Center for Tea Improvement, 1 Hu Tou Yang Road, She Kou, Fu An, 355015 Fu Jian China

**Keywords:** Tea cultivar, SSR markers, Fingerprinting, Phylogenetic analysis, Cultivar identification diagram (CID)

## Abstract

Microsatellites, or simple sequence repeats (SSRs), especially those with long-core motifs (tri-, tetra-, penta-, and hexa-nucleotide) represent an excellent tool for DNA fingerprinting. SSRs with long-core motifs are preferred since neighbor alleles are more easily separated and identified from each other, which render the interpretation of electropherograms and the true alleles more reliable. In the present work, with the purpose of characterizing a set of core SSR markers with long-core motifs for well fingerprinting clonal cultivars of tea (*Camellia sinensis*), we analyzed 66 elite clonal tea cultivars in China with 33 initially-chosen long-core motif SSR markers covering all the 15 linkage groups of tea plant genome. A set of 6 SSR markers were conclusively selected as core SSR markers after further selection. The polymorphic information content (PIC) of the core SSR markers was >0.5, with ≤5 alleles in each marker containing 10 or fewer genotypes. Phylogenetic analysis revealed that the core SSR markers were not strongly correlated with the trait ‘cultivar processing-property’. The combined probability of identity (PID) between two random cultivars for the whole set of 6 SSR markers was estimated to be 2.22 × 10^−5^, which was quite low, confirmed the usefulness of the proposed SSR markers for fingerprinting analyses in *Camellia sinensis*. Moreover, for the sake of quickly discriminating the clonal tea cultivars, a cultivar identification diagram (CID) was subsequently established using these core markers, which fully reflected the identification process and provided the immediate information about which SSR markers were needed to identify a cultivar chosen among the tested ones. The results suggested that long-core motif SSR markers used in the investigation contributed to the accurate and efficient identification of the clonal tea cultivars and enabled the protection of intellectual property.

## Background

Tea produced from fresh leaves of the tea plant *Camellia sinensis* (L.) O. Kuntze, is used worldwide. Its attractive aroma, flavor, and medicinal benefits are derived from compounds such as polyphenols, caffeine, and amino acids (Mejia et al. 2009; Sharangi 2009). Tea plant is a woody evergreen plant of the genus *Camellia* belonging to the family Theaceae, which has been cultivated in more than fifty countries including Asia, Africa, South America, Europe, Oceania, and contributed to massive economic development in these areas (Anesini et al. 2008; Alkan et al. 2009; Basu Majumder et al. 2010; Sae-Lee et al. 2012). In 2013, 3.52 million hectares of tea plants were harvested, producing 5.34 million tons of tea (FAO, http://faostat.fao.org/). The clonal tea cultivars are characterized by a regular and uniform development of shoots and leaves period, leading to a stable tea quality, and improved tea yield (Wachira et al. 1995; Fang et al. 2012; Yao et al. 2011). In recent years, tea acreage and production have increased continuously, partially as a result of the release and extension of clonal tea cultivars (Bandyopadhyay 2011).

Tea plant is a woody perennial characterized by a large diploid genome (~4 Gb, 2n = 30, very few are triploid), which has not been sequenced so far. It is self-incompatible and highly heterozygous. It has a long juvenile phase (more than 20 years), therefore tea cultivar breeding is a very long and expensive process (Chen et al. 2007; Tan et al. 2013). Tea tree is capable of multiplying by vegetative propagation of its shoots, as a result, the phenomenon of infringement of clonal tea cultivar breeders’ rights is extremely common. Therefore, to safeguard the protection of intellectual property, it is crucial to establish a fast, scientific, and practical method to identify them.

The traditional method of morphological identification failed to effectively identify several clonal tea cultivars, due to the effect of environmental factors on phenotypic traits. By contrast, DNA molecular markers have proved to be a powerful tool for fingerprinting of crop cultivars (Patzak et al. 2007; Jian et al. 2010; Divashuk et al. 2011). SSR markers are characterized by codominance, polymorphism, and high stability, and therefore, represent a superior choice among all the molecular markers developed for crop cultivar identification (Hasnaoui et al. 2012; Karaagac et al. 2014).

Recent advances in SSR for tea came from the deep sequencing of the tea plant transcriptome (Wu et al. 2012; Tan et al. 2013; Wang et al. 2013), which provided an increased number of SSR markers for tea cultivar identification. Several studies have investigated tea cultivars with SSR markers (Kaundun and Matsumoto 2004; Ujihara et al. 2009; Bhardwaj et al. 2013), nevertheless, these studies would be not so straightforward but they would be done using a little more time to analyze the fingerprinting data in discriminating tea cultivars. With taking advantage of the suggested necessary SSR markers, a pratical strategy for efficient identification of plants rely on a new way of recording DNA fingerprints of genotyped plants called cultivar identification diagram (CID), which can be used for a quick identification of specific plant cultivars (Huo et al. 2013). In view of this, the CID method can be used as a practical way in identifying clonal tea cultivars.

The fingerprinting data should be supposed to repeatedly, so the accuracy of which were of great importance. SSR with long-core motifs (tri-, tetra-, penta-, and hexa-nucleotide) are preferred since neighbor alleles are more easily separated and identified from each other. Short-core motifs (di-nucleotide) are not desirable mainly because of the lower separation of neighbor alleles and the high degree of stuttering, which render the interpretation of electropherograms and the true alleles less reliable (Cipriani et al. 2008). SSR with long-core motifs were adopted in human genetics (Ruitberg et al. 2001; Butler et al. 2004; Butler 2006; Hellmann et al. 2006), but were exclusively used for genetic analyses only in few crops (Dettori et al. 2015). In tea plant, there have been reported that eight core SSRs with the larger repeat motifs (3–6 bp) selected to fingerprint 128 Chinese clonal tea cultivars (Tan et al. 2015), nevertheless, it would require a little more time to analyze the fingerprinting data using these SSR markers in discriminating tea cultivars, furthermore, these markers would be not enough to exclusively identify tea cultivars, especially when more new cultivars would be released in future.

Therefore, for the sake of providing a practical method of identification of the clonal tea cultivars, thus ensuring the protection of intellectual property, we aimed to obtain a new set of long-core motif SSR markers, and to establish a cultivar identification diagram (CID) based on the suggested necessary SSR markers and the genotyping data revealed, fully reflecting the identification process and providing the immediate information about which SSR markers are needed to identify a cultivars chosen among the tested ones.

## Methods

### Plant material

A total of 66 elite clonal tea cultivars were tested in this study. They were collected during the tea germplasm collection at the Institute of Tea, Fujian Academy of Agricultural Sciences, Fuan, Fujian, China. Young leaves of the 66 clonal tea cultivars were collected twice independently and frozen in liquid nitrogen, and stored at −80 °C. The names of these clonal tea cultivars are listed in Table 1.

**Table 1:** Information of the tested clonal tea cultivars

Code	Cultivar name	Area of origin	CPP^a^	Code	Area of origin	Area of origin	CPP^a^
1	Tie Guan Yin	Fu Jian	O	34	Yue Min Xiang	Fu Jian	O/G/B
2	Huang Dan	Fu Jian	O	35	Huang Qi	Fu Jian	O/G/B
3	Ben Shan	Fu Jian	O	36	Jin Mu Dan	Fu Jian	O/G/B
4	Mao Xie	Fu Jian	O	37	Huang Mei Gui	Fu Jian	O/G/B
5	Mei Zhan	Fu Jian	O	38	Zi Mu Dan	Fu Jian	O/G/B
6	Feng Yuan Chun	Fu Jian	O	39	Zi Mei Gui	Fu Jian	O/G/B
7	Xing Ren Cha	Fu Jian	O	40	Zao Chun Hao	Fu Jian	G/B
8	Hong Ya Fo Shou	Fu Jian	O	41	Chao Yang	Fu Jian	O/G/B
9	Lv Ya Fo Shou	Fu Jian	O	42	Dan Gui	Fu Jian	O/G/B
10	Da Ye Wu Long	Fu Jian	O	43	Chuan Lan	Fu Jian	O/G/B
11	Bai Ya Qi Lan	Fu Jian	O	44	Rui Xiang	Fu Jian	O/G/B
12	Ba Xian Cha	Fu Jian	O	45	Jiu Long Pao	Fu Jian	O/G/B
13	Da Hong Pao	Fu Jian	O	46	Chun Gui	Fu Jian	O/G/B
14	Rou Gui	Fu Jian	O	47	Zao Mei Gui	Fu Jian	G/B
15	Bai Ji Guan	Fu Jian	O	48	Ming Ke 3	Fu Jian	G/B
16	Ai Jiao Wu Long	Fu Jian	O	49	Ming Ke 4	Fu Jian	G/B
17	Fu Jian Shui Xian	Fu Jian	O	50	Chun Tao Xiang	Fu Jian	O/G/B
18	Zheng He Da Bai Cha	Fu Jian	G/B	51	Jin Mei Gui	Fu Jian	O/G/B
19	Jiu Long Da Bai Cha	Fu Jian	G/B	52	Zi Guan Yin	Fu Jian	O/G/B
20	Fu Ding Da Bai Cha	Fu Jian	G/B	53	Jin Gui Guan Yin	Fu Jian	O/G/B
21	Fu Ding Da Hao Cha	Fu Jian	G/B	54	Zhong Cha 108	Zhe Jiang	G/B
22	Ge Le Cha	Fu Jian	G/B	55	Wu Niu Zao	Zhe Jiang	G/B
23	Fu An Da Bai Cha	Fu Jian	G/B	56	Yin Shuang	Zhe Jiang	G/B
24	Xia Pu Chun Bo Lv	Fu Jian	G/B	57	An Ji Bai Cha	Zhe Jiang	G/B
25	Xia Pu Yuan Xiao Cha	Fu Jian	G/B	58	Long Jin 43	Zhe Jiang	G/B
26	Rong Chun Zao	Fu Jian	G/B	59	Qian Nian Xue	Zhe Jiang	G/B
27	Fu Yun 6	Fu Jian	G/B	60	Ping Yang Te Zao	Zhe Jiang	G/B
28	Fu Yun 7	Fu Jian	G/B	61	Li Zao Xiang	Zhe Jiang	G/B
29	Fu Yun 10	Fu Jian	G/B	62	Si Ji Chun	Tai Wan	O
30	Fu Yun 20	Fu Jian	G/B	63	Jin Xuan	Tai Wan	O
31	Fu Yun 595	Fu Jian	G/B	64	Bai Mao 2	Guang Dong	O/G/B
32	Jin Guan Yin	Fu Jian	O/G/B	65	Feng Huang Dan Cong	Guang Dong	O
33	Huang Guan Yin	Fu Jian	O/G/B	66	Xiang Fei Cui	Hu Nan	G/B

^a^ *CPP* cultivar processing-property, indicated one cultivar is suitable for processing one type/different types of tea. *O* Oolong tea, *G* green tea, *B* black tea

### DNA extraction

Total genomic DNA of each cultivar was extracted twice from young leaves using the CTAB method (Reitz et al. 1972). The genomic DNA was diluted to a final concentration of 30 ng/μL using TE buffer and stored at −20 °C until use, and 0.8 % agarose gels were used to check the quality of the DNA.

### PCR and SSR fragment detection

The 33 SSR markers with long-core motifs (tri-, tetra-, penta-, hexa-nucleotides) from a tea plant genetic map (Ma et al. 2014), were initially selected and labeled at the 5′ end of each forward primer pair with fluorescent dyes. To the best of our knowledge, this set of markers has not been used in identifying tea cultivars so far. The selection criteria were follows: (1) two or more alleles detected in a preliminary screening with eight cultivars (data not shown); (2) alleles distinct on 10 % silver-stained polyacrylamide gels with no random bands; and (3) markers with an even coverage of 15 linkage groups of tea plant genome. The details about the markers used in this study were showed in Table 2.

**Table 2:** 33 SSR markers selected from 15 linkage groups of tea plant

Linkage	Primer	Motif	Forward (5′ → 3′)	Reverse (5′ → 3′)	Tm (°C)	Dye
LG01	TM447	(AAAAG)5	TGTTGTTAACGGTGTTCGGA	GCATTTGTTTTCTCTCTCTGCC	52	TAMRA
LG02	TM514	(TCA)5	ATGTCTGGCCGTGGATTAAG	ATGGCAGGCTGTTCTGATTT	52	FAM
LG02	TM480	(GTA)5	CGAAGAGTCGTTTCGAGGAG	CATCCCTTGTCTTCTCCCCT	52	FAM
LG03	TM337	(CCAATT)6	GTGCGGCAAAGCTGTCTT	ACCTCCATCTCCAAACCC	60	FAM
LG03	TM453	(TTC)6	AAGTCACAACACCACCACCA	GAGGCAGCGATAGTACCAGG	52	TAMRA
LG04	TM343	(TGTTGA)3	ATCTTGGTAAGCTGCTCT	ATCATTGCTTTTGTTCTG	56	FAM
LG04	TM445	(GTA)5	CCCAAATCCCAAGCTGTAGA	ACGATCGAGCCTGCAATACT	52	TAMRA
LG04	TM502	(AGAT)4	TGTCTTTTGTGGTTTCGTGC	GGGAGACGATGGATCAGAAA	50	FAM
LG04	TM422	(TTC)7	GGACTTCGTTGCTTCCTTTG	CCATTCTCGACGAATCCAGT	52	TAMRA
LG04	TM369	(GAA)8	CGGAGCTGGAATCTGAAGAG	GGAAGGGTTGCAAATTCTGA	52	FAM
LG04	TM523	(AAAAGA)3	TTTGCATTTTTGCCAAGTGA	CTTGCGTGACAATGCTCATAA	52	FAM
LG05	TM589	(CTCCT)3	CACCACTGCCCAACAAACT	GAGGATGATGATTCGGGAGA	52	TAMRA
LG05	TM428	(CAC)7	TCTCCTCCTCGATCCTCAGA	CCCTCTTCTTCGGATCCTTC	52	FAM
LG06	TM341	(TCGAA)5	CGTACTTCAACGCTATAGCTCTCT	CTTCGGCATGGCTTCTAAAC	52	FAM
LG07	TM415	(CCTTC)3	TCCACCCAAAACCTACTCTCTC	TATTTCGGAAACGAGCCATC	52	TAMRA
LG07	TM426	(AGA)11	TGAGAGTGCTTGTCTGGGTG	CAACTACCCCTTTTCCCCAT	52	FAM
*LG07* ^a^	*TM324*	*(TTTTTG)5*	*CATCGTTTCATTGCTTATT*	*ATTTTCGGCATTGTCTT*	*54*	*FAM*
LG08	TM352	(GAGGTG)4	CTTCTTCCTGTCGGGTTGAG	GTCAACGGCCTATAACGGAA	52	TAMRA
LG08	TM395	(TCTTTT)4	GATTGTAGGACAGCCGTGGT	AAGTTGGGGCTTGTTAAAGGA	52	FAM
LG08	TM493	(AGG)6	GATAGGGACAGAGATCGGCA	TTTCCAACCTTGCTCAAACC	52	FAM
*LG09* ^*a*^	*TM442*	*(ATACAC)3*	*CAAGCCAAACCTTGCTGAAT*	*CTGTCCTGTGTCTGGTGGTG*	*52*	*FAM*
LG09	TM440	(TTTGC)3	TTGACCCGAATAAAATGGGA	CCTCAAAACATGCTTTTCTTAATC	52	FAM
LG10	TM407	(CAAGAT)3	AACAACAGCAGCGAAGATGA	CCACCACTGATGACCCTTTT	52	TAMRA
*LG10* ^*a*^	*TM569*	*(GTGA)5*	*GCAAATTCGTAAGGCGAGAG*	*CTGACGTTTACCCTCGTTCC*	*52*	*FAM*
*LG11* ^*a*^	*TM461*	*(ATTTTT)6*	*GGCTAGGGTTTCTCCCACTT*	*GAAGGTCGAAGCGATGTTGT*	*52*	*TAMRA*
*LG11* ^*a*^	*TM581*	*(AAAAAC)3*	*AAGGATCACTGGTAAAAAGCCA*	*CTTCTGAGCCGTTCTTGAGC*	*52*	*FAM*
LG12	TM241	(GAGAA)3	ATCGGCGACGGTGGAAGT	GCCAGCGGAGAGGAGAAG	58	FAM
LG12	TM499	(AGA)5	AACTGTGACACCGATTGCAG	AAGTTTCACTTGCCAGCACC	54	FAM
LG13	TM425	(TTATT)3	CACGTTCGCATATTTTGGTG	TTGCTGACGACAACATTTTATT	52	FAM
LG13	TM576	(TTTTC)3	CGCTCTTCCTTGTTTTCTGG	CACAAGCCATTGTAGAGAGAGAAA	52	FAM
LG14	TM348	(TATC)7	GAGATGGCTTGCTCAAGGTC	CCCCAACCAAATCAAATCAC	52	FAM
*LG14* ^*a*^	*TM351*	*(GGAGAA)3*	*GGGTGAGAGTAAAGGGGGAG*	*AAACACAAAATCAAATTTGTCAGAA*	*52*	*FAM*
LG15	TM601	(GGA)5	TTGCACTGGAGTGCGATAAG	CATCGCCACCAAACTCTTCT	52	FAM

^a^Selected as core SSR markers to fingerprint 66 clonal tea cultivars

PCR amplification was carried out in a volume of 30 μL, containing 2 μL of (30 ng/μL) genomic DNA, 1.5 μL (10 μM) of each primer, 1.0 μL of Taq DNA polymerase (0.5 U/μL), 2 μL (25 mM) of MgCl_2_, 3 μL dNTP (10 mM), 3 μL 10 × Buffer, and 16 μL of double-distilled water. Amplification reactions were performed using Huayue Biometra Thermal Cycler under the following conditions: initial denaturation for 5 min at 94 °C, 35 cycles at 94 °C for 30 s, Ta °C for 30 s, and at 72 °C for 1 min, and a final extension step at 72 °C for 20 min plus a hold at 4 °C. The Tm (°C) of each primer was reported in Table 2. After mixing 1µL of each PCR product with 9 µL of the standard molecular weight mixture ROX500 in a 96-well-plate, it was gently vortexed, and centrifuged at 3000 rpm for 2 min. The mixture was denatured at 95 °C for 3 min and left in ice for 5 min, and loaded into the Applied Biosystems (ABI) 3730 sequencer for fragment analysis. Both PCR amplification and SSR fragment detection were performed twice independently.

### Data analysis

The data obtained were analyzed using Genemapper software version 4.0. PowerMarker (Liu and Muse 2005) was used to calculate the key genetic statistics of the markers, including major allele frequency (MAF), number of alleles (NA), number of genotype (NG), observed heterozygosity (Ho), polymorphism information content (PIC), and Nei’s genetic distances (Nei et al. 1983).

### Power of fingerprinting

To assess the fingerprinting potential of the SSR markers, probability of identity (PID) for each marker was calculated. PID represents the average probability of two random individuals in a population sharing the same genotype, and is calculated as follows:

$${\text{PID}} = 2(\varSigma {\text{P}}_{\text{i}}^{2} )^{2} - \varSigma {\text{p}}_{\text{i}}^{4} ,$$

where p_i_ is the frequency of the ith allele at a locus (Taberlet and Luikart 1999).

### Core SSR marker selection

Additional criteria used for selection of core SSR markers include: (1) PID < 0.198 (average of the 33 markers), such that a combination of a few markers provided enough discriminant power; (2) high degree of polymorphism, with a PIC > 0.5 (Hoda et al. 2010; Pan et al. 2010); and (3) finally, the number of alleles ≤5, and the number of genotypes ≤10. Primers providing higher numbers of alleles and of genotypes were not chosen because they were not deemed to be easily manageable.

### Phylogenetic analysis

For represented all the variability in the tested tea cultivars, using the set of core SSR markers selected, a phylogeny tree of the tested clonal tea cultivars was constructed based on Nei’s genetic distances and the UPGMA method and viewed with MEGA 4.0 (Tamura et al. 2007).

### Construction of CID

The CID was established as reported previously (Liu et al. 2014) with a few modifications. The method was based on classification of cultivars into different groups according to the genotypes amplified by each core SSR marker selected: (1) a cultivar with a unique genotype generated from a single primer pair, was already identified and occupied a group by itself; (2) cultivars sharing the same genotype were placed in the same group; (3) additional core SSR marker primer pairs were used to identify the cultivars sharing the same group; and (4) the order of core markers selected to construct the CID was chosen at descending PID values.

## Results

### Stability of detection and data analysis

The allele size was the same in the two independent scoring, which inicated the high quality and stability of the DNA fragments amplification and detection. All the 33 SSR loci were polymorphic among the tested cultivars. The NA ranged from 2 to 16 (average 6), and the NG ranged from 2 to 33 (average 11.2). The Ho ranged from 0.136 to 0.864 (average 0.583), and the PIC ranged from 0.119 to 0.864 (average 0.553). Details are displayed in Table 3. The average values of NA, Ho and PIC were all lower than those of Tan reported (Tan et al. 2015), where NA, Ho and PIC was 10.4, 0.701, 0.704, respectively, which mainly owing to the 33 SSR markers used herein were all long-core motif ones.

**Table 3:** Key genetic statistics of the 33 SSR markers

Linkage group	Primer	MAF	NG	NA	Ho	PIC	PID
LG01	TM447	0.470	8	4	0.470	0.510	0.226
LG02	TM514	0.796	5	4	0.273	0.286	0.415
LG02	TM480	0.660	8	6	0.515	0.481	0.222
LG03	TM337	0.409	20	9	0.758	0.722	0.075
LG03	TM453	0.553	15	8	0.561	0.562	0.157
LG04	TM343	0.697	11	9	0.561	0.475	0.115
LG04	TM445	0.432	7	4	0.515	0.554	0.203
LG04	TM502	0.508	13	6	0.727	0.655	0.095
LG04	TM422	0.220	33	16	0.864	0.864	0.023
LG04	TM369	0.424	18	10	0.773	0.706	8.58E-07
LG04	TM523	0.811	5	3	0.227	0.297	0.454
LG05	TM589	0.477	6	3	0.606	0.550	0.199
LG05	TM428	0.386	17	7	0.727	0.720	0.080
LG06	TM341	0.356	23	12	0.606	0.765	0.048
LG07	TM415	0.288	26	13	0.530	0.801	0.040
LG07	TM426	0.280	17	7	0.652	0.761	0.076
*LG07* ^*a*^	*TM324*	*0.402*	*7*	*4*	*0.636*	*0.577*	*0.185*
LG08	TM352	0.674	5	3	0.546	0.431	0.244
LG08	TM395	0.660	5	3	0.591	0.432	0.274
LG08	TM493	0.576	10	7	0.682	0.585	0.123
*LG09* ^*a*^	*TM442*	*0.523*	*9*	*4*	*0.485*	*0.560*	*0.192*
LG09	TM440	0.432	14	6	0.864	0.683	0.100
LG10	TM407	0.667	15	10	0.515	0.527	0.124
*LG10* ^*a*^	*TM569*	*0.386*	*7*	*4*	*0.788*	*0.594*	*0.180*
*LG11* ^*a*^	*TM461*	*0.296*	*10*	*5*	*0.773*	*0.685*	*0.115*
*LG11* ^*a*^	*TM581*	*0.538*	*9*	*4*	*0.621*	*0.580*	*0.165*
LG12	TM241	0.417	11	4	0.788	0.650	0.136
LG12	TM499	0.758	5	3	0.439	0.338	0.345
LG13	TM425	0.932	2	2	0.136	0.119	0.645
LG13	TM576	0.833	3	3	0.333	0.245	0.441
LG14	TM348	0.379	12	5	0.712	0.660	0.123
*LG14* ^*a*^	*TM351*	*0.568*	*9*	*5*	*0.576*	*0.549*	*0.183*
LG15	TM601	0.773	5	4	0.394	0.337	0.338
*Mean*		*0.533*	*11.2*	*6.0*	*0.583*	*0.553*	*0.198*

^a^Selected as core SSR markers to fingerprint 66 clonal tea cultivars

### Selection of core SSR markers

According to the above selection criterion, 6 SSR markers with long-core motifs were further selected from the 33 SSR markers, and used as a set of core primer pairs to identify the clonal tea cultivars tested (Tables 2, 3).

### Power of fingerprinting

For each locus, the PID ranged from 8.58E-07 to 0.645, averaging 0.198 (Table 3). Assuming that all loci segregate independently, the probability of finding two random individuals with identical genotypes at all the 33 loci was an estimated 1.42 × 10^−32^, while the probability of the set comprising 6 core SSR markers was 2.22 × 10^−5^, which provided enough discriminant power to identify the tested clonal tea cultivars.

### Genotypes of the tested cultivars

The genotypes of the 66 cultivars generated from the set of 6 core SSR markers are shown in Table 4, by which we can establish the CID to rapidly distinguish the tested clonal tea cultivars.

**Table 4:** Core set of six primers with genotypes of 66 cultivars

Code^a^	TM442	TM324	TM351	TM569	TM581	TM461
1	268/292	171/183	244/244	265/269	228/234	192/210
2	286/286	183/183	244/250	265/273	228/228	192/198
3	268/280	171/183	244/250	265/269	228/228	198/198
4	280/280	171/183	244/250	269/273	228/234	192/198/204
5	280/280	171/183	244/244	265/269	228/234	192/198
6	268/280	171/183	238/244	265/265	228/234	198/198
7	280/280	177/183	244/244	265/269	234/234	192/198
8	268/280	171/171	238/244	265/269	228/228	192/204
9	268/280	171/171	244/244	265/269	222/228	192/198
10	268/280	171/171	238/244	269/269	228/228	192/204
11	280/280	171/177	238/244	269/273	228/234	192/204
12	280/286	183/183	238/238	265/269	222/228	192/204
13	268/280	177/183	238/262	265/273	228/234	204/204
14	268/280	171/177	244/244	269/273	228/228	192/204
15	292/292	183/183	238/244	273/273	222/228	204/204
16	280/280	171/177	244/244	269/273	228/228	192/198
17	280/286	171/183	244/256	265/277	228/234	192/198
18	286/286	171/177/183	238/250	265/269/273	228/234	198/204/210
19	280/280	183/183	238/244	269/273	228/228	204/210
20	268/280	171/183	244/244	269/273	222/228	204/210
21	280/280	183/183	238/244	269/273	228/228	186/210
22	268/280	171/183	244/244	273/273	222/228	204/210
23	280/286	183/183	244/256	269/273	222/228	204/204
24	280/286	183/183	244/250	265/269	228/228	198/210
25	280/286	171/183	238/238	269/273	222/222	204/210
26	280/286	183/183	238/250	269/273	222/228	192/198
27	280/280	177/183	244/244	265/269	228/240	198/204
28	280/280	162/171	244/250	273/273	222/240	198/204
29	280/280	171/183	244/250	269/273	228/228	204/210
30	280/280	171/171	244/256	269/273	228/240	198/204
31	280/280	177/183	244/250	273/273	222/240	198/210
32	280/286	171/177	244/250	265/273	228/234	192/192
33	286/286	177/183	244/250	265/269	222/228	192/204
34	268/280	171/171	244/244	269/273	228/228	192/204
35	286/286	183/183	244/244	269/273	228/228	192/192
36	280/286	171/177	244/244	265/269	222/228	198/198
37	286/286	177/183	250/250	265/265	222/228	192/204
38	280/286	171/171	244/256	265/273	228/240	198/204
39	280/286	171/177	244/244	265/269	228/234	192/192
40	268/268	171/177	250/250	265/269	222/234	204/210
41	280/292	171/177	248/250	269/273	222/240	198/198
42	280/286	171/183	244/244	273/273	228/228	204/204
43	286/286	171/183	244/244	269/269	228/228	192/198
44	280/286	177/183	244/244	269/273	228/228	192/198
45	268/280	177/177	244/250	269/269	228/234	198/204
46	286/286	177/183	244/244	269/273	228/228	192/192
47	280/286	177/183	244/244	265/269	228/228	192/204
48	280/280	177/183	250/250	265/273	240/240	198/204
49	280/280	171/177	244/250	269/273	222/228	204/204
50	286/286	171/183	244/244	265/269	222/228	198/204
51	280/286	171/171	244/250	265/269	228/234	192/198
52	286/286	177/183	244/250	269/273	228/234	192/198
53	280/286	171/171	244/250	265/269	228/234	192/192
54	280/280	171/183	244/250	265/269	222/234	198/210
55	268/280	183/183	250/256	265/269	222/222	192/204
56	268/286	171/171	244/244	265/273	222/228	204/210
57	280/280	177/183	244/256	273/273	228/228	198/204
58	280/280	171/177	244/256	273/273	228/228	198/210
59	286/286	183/183	244/244	265/269	222/234	198/210
60	268/280	183/183	250/256	265/273	222/240	192/198
61	280/280	177/183	250/250	265/265	222/222	198/210
62	280/280	171/177	244/244	269/273	228/234	192/204
63	280/280	171/171	244/250	269/273	228/234	198/204
64	280/292	171/177	244/250	265/273	228/240	204/210
65	268/286	171/183	244/250	269/269	222/234	198/198
66	268/268	177/183	250/256	265/269	234/234	192/210

^a^The 66 codes are in accord with those in Table 1

### Phylogenetic analysis

**Fig. 1:**
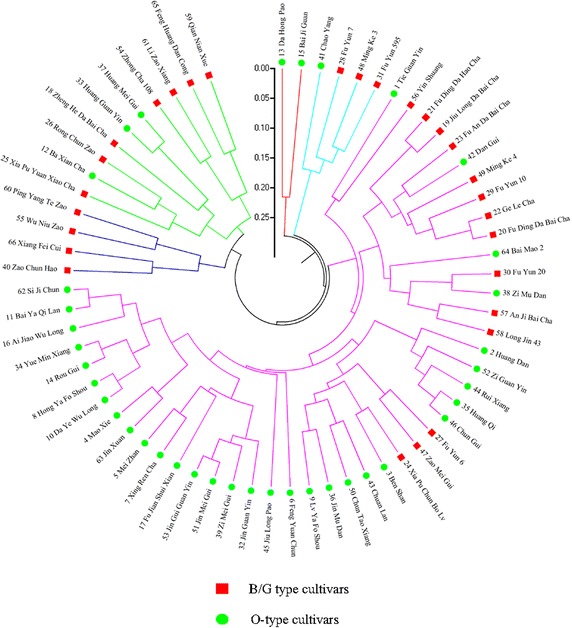
Phylogeny tree showing the clustering pattern of the 66 tested clonal tea cultivars. O-type cultivars: cultivars suitable for processing oolong tea; B/G-type cultivars: cultivars suitable for processing black tea/green tea

The genetic relationships among the tesrted 66 tea cultivars are presented in the phylogeny tree (Fig. 1). All of them were grouped according to their genetic backgrounds. To better understand their relationships, we divided the tested cultivars into five groups according to the genetic distance value at 0.25. The first cluster was comprised of two cultivars, including 13th (Da Hong Pao) and 15th (Bai Ji Guan). The second cluster included four cultivars, including 41st (Chao Yang), 28th (Fu Yun 7), 48th (Ming Ke 3), 31st (Fu Yun 595). The fourth cluster also included 4 cultivars, including 40th (Zao Chun Hao), 66th (Xiang Fei Cui), 55th (Wu Niu Zao), 60th (Ping Yang Te Zao). The fifth cluster included 10 cultivars, including 25th (Xia Pu Yuan Xiao Cha), 12th (Ba Xian Cha), 26th (Rong Chuan Zao), 18th (Zheng He Da Bai Cha), 33th (Huang Guan Yin), 37th (Huang Mei Gui), 54th (Zhong Cha 108), 61st (Li Zao Xiang), 65th (Feng Huang Dan Cong), 59th (Qian Nian Xue). The third cluster was comprised of the maximum number of materials, including the rest 46 ones of the tested cultivars. From the phylogeny tree, we could find the clustering result based on the genetic background (Nei’s genetic distances) was not well corresponded with that based on the trait ‘cultivar processing-property’. This phenomenon showed that the core SSR markers were not strongly correlated with the trait ‘cultivar processing-property’.

### Creation of CID

**Fig. 2:**
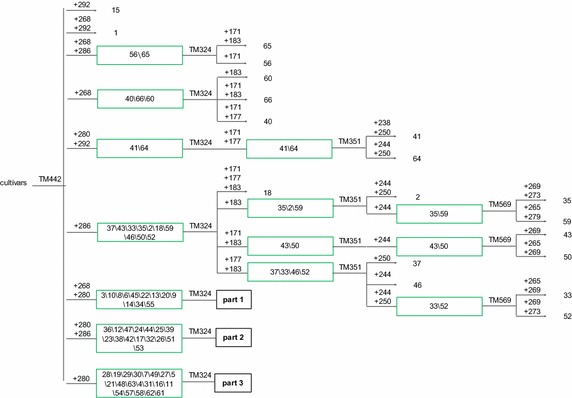
Cultivar identification diagram (CID) of clonal tea cultivars (I). The codes are in accord with those in Table 1

**Fig. 3:**
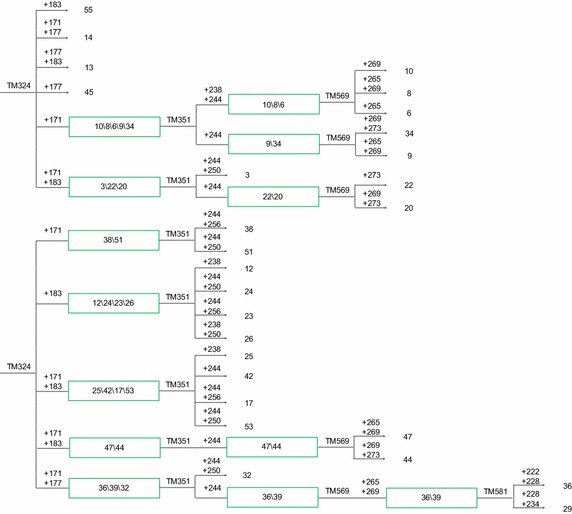
Cultivar identification diagram (CID) of clonal tea cultivars (II). The codes are in accord with those in Table 1

**Fig. 4:**
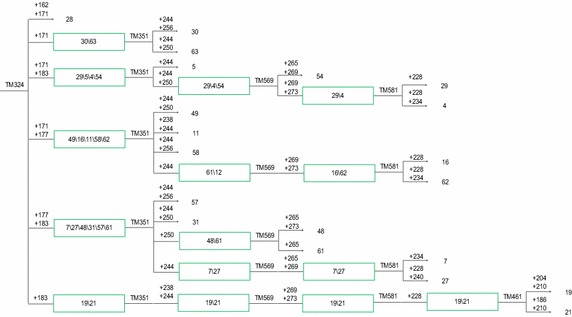
Cultivar identification diagram (CID) of clonal tea cultivars (III). The codes are in accord with those in Table 1

Based on the descending order of PID values of the six core markers, the primer pair with the highest PID, TM442 (PID = 0.192), was used to identify the genotypes of all the tested cultivars. It allowed to classify the cultivars into nine groups corresponding to the nine genotypes. The first group contained only the 15th cultivar (Bai Ji Guan, genotype ‘+292’), and the second group contained only the first cultivar (Tie Guan Yin, genotype ‘+268,292’). The remaining 64 cultivars were distributed into seven groups, containing each more than two cultivars, requiring other markers for separation. The third group including two clonal tea cultivars, namely, the 56th (Yin Shuang) and the 65th (Feng Huang Dan Cong), which were separated by the genotype generated by the second primer pair TM324 (PID = 0.185), ‘+171’ and ‘+171, 183’, respectively. Similarly, the other groups of clonal tea cultivars were fully separated by other primer pairs, and the CID was established as shown in Figs. 2, 3 and 4.

**Fig. 5:**
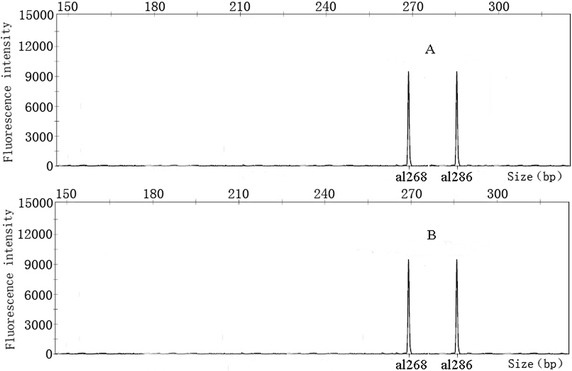
Fingerprinting mode of two clonal tea cultivars genotyped by TM442. The genotypes of the two cultivars were both ‘+268, +286’

**Fig. 6:**
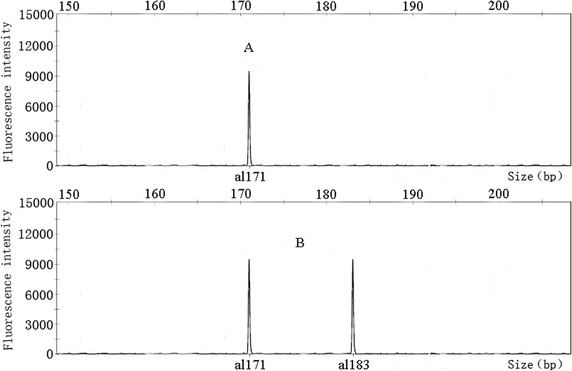
Fingerprinting mode of two clonal tea cultivars genotyped by TM324. The genotype of ‘A’ cultivar was ‘+171’, The genotype of ‘B’ cultivar was ‘+171, +183’

Being the information about markers used contained in the CID, it can make the identification of the cultivars represented more straightforward. In the following example two random cultivar, A and B, belonging to the CID, were screened by TM442, the first primer used in CID construction. The genotypes of the two cultivars were both ‘+268, +286’, placing them on the third group of the CID. After that, TM324 showed that ‘A’ genotype was ‘+171’, and ‘B’ was ‘+171, +183’ thus identifying ‘A’ as the 56th(Yin Shuang), and ‘B’ as the 65thcultivar (Feng Huang Dan Cong). Details are shown in Figs. 5 and 6. Using this procedure, the two cultivars were quickly and successfully identified with the suitable combination of two primers (TM442 and TM324).

Upon release of new clonal tea cultivars, the set of six core SSR markers could still be used to amplify their genomes and locate them on the CID; furthermore, this could be achieved using less than the six primers currently included in the core set. For example, if a new cultivar displayed the TM442 ‘+268, 292’ genotype and TM324 was ‘+183’, the CID construction or the identification process could be finished using only two primer pairs. On the contrary, if the six primer pairs would fail to provide a full identification of the new cultivars, additional core SSR primer pairs could be added. With the identification of new cultivars, a larger clonal tea cultivar CID would be developed.

## Discussion

Recently the single nucleotide polymorphism (SNP) has rapidly become a well-considered marker choice for genetic studies, due to their low cost, high genotyping efficiency, genome-wide coverage and analytical simplicity. SNP markers have been used in tea plant for cultivar identification (Fang et al. 2014), genetic diversity analysis (Yang et al. 2016), genetic map construction (Ma et al. 2015), nevertheless, it does not mean we cannot use SSR markers anymore. SSRs are still served as excellent markers in tea plant genetic analyses. As useful genetic markers, SSRs have been provided with several advantages for their co-dominant, hyper-variability, polymorphism, ease and reliability of scoring. SSRs have been used extensively for analysis of genetic diversity, population genetics, linkage mapping and association analysis (Verma et al. 2012). Furthermore, the high PIC value of SSRs (up to three fold higher than SNPs), coupled with high heterozygosity values makes them useful for assessment of genetic relatedness and map based cloning (Yang et al. 2011). We reported the combined PID value of only 6 long-core motif SSRs herein was 2.22 × 10^−5^, while that of 60 polymorphic SNPs was about 1.0 × 10^−5^ (Fang et al. 2014). That was to say, the discriminating power of only 6 long-core motif SSRs reached approximately half to that of 60 SNPs. In comparison, the advantage of SSRs over SNPs for individual fingerprinting is obvious.

The successes of DNA fingerprinting greatly rely on the markers’ quality and the accuracy of genotyping data, thus supposed to be perfectly repeatable in every lab. In the present work, a great deal of attention have been paid to the marker selection step. The 33 markers initially selected from a tea plant genetic map were all long-core motif ones, which render the interpretation of electropherograms and the true alleles more reliable. Furthermore, owing to additional criteria used for selection, the new set of 6 core SSR markers were all provided with enough discriminant power, and were highly polymorphic, easily manageable, which helped in improving the identification efficiency. The capillary electrophoresis conducted in ABI 3730 sequencer was capable of providing high detection sensitivity of amplified DNA fragments, which has been proved to be a powerful and efficient technique for automated and accurate estimation of allele sizes (Brunings et al. 2010; Li et al. 2014). There were no differences between the two independent replicates, which both detected by the capillary electrophoresis technology, showed the detection results held stability and reproducibility. The set of 6 core SSR markers in this investigation were valuable resources, and were of great importance in tea cultivar fingerprinting.

The phylogeny tree was incapable of providing us which information could be used for the identification of the tested cultivars, although it represented all the variability in the tested cultivars. By using the genotypes of the tested cultivars (Table 4 showed), everyone could decide which primers to use to identify two cultivars, nevertheless, it would be not so straightforward but it would be done using a little more time to analyze the fingerprinting data in discriminating the tested tea cultivars. The CID directly allowed separation of cultivar sample at each step, and the whole identification process was displayed, which differed from the phylogeny tree and was an extension of the fingerprinting data. For these reasons, the CID method was a kind of useful complements to the phylogeny tree and fingerprinting data when used to quickly identify tea cultivars.

From the phylogeny tree, we can find that the core SSR markers are not strongly correlated with the trait ‘cultivar processing-property’. In the near future, we will focus on the study of the linkage disequilibrium (LD)-based association analysis (Gupta et al. 2005) through SSR scanning of diverse tea cultivars (or germplasms), to detect SSR markers strongly correlated with target traits to help marker assistant selection in tea-breeding programs.

## Conclusions

The 6 core SSR markers with long-core motif selected in the study on the bases of the degree of gene polymorphism and of genotype frequencies revealed, of easy and stable allele separation and scoring, enabled the full identification of 66 tested clonal tea cultivars.

The tea plant CID based on the suggested core SSR markers and genotyping data revealed, was a useful complement to the phylogeny tree and the fingerprinting data, provides help in quickly identifying the clonal tea cultivars and, consequently, in protecting the plant breeders’ rights.

## References

[CR1] Alkan I, Köprülü O, Alkan B (2009). Latest advances in world tea production and trade, Turkey’s aspect. World J Agric Sci.

[CR2] Anesini C, Ferraro GE, Filip R (2008). Total polyphenol content and antioxidant capacity of commercially available tea (*Camellia sinensis*) in Argentina. J Agric Food Chem.

[CR3] Bandyopadhyay T (2011). Molecular marker technology in genetic improvement of tea. Int J Plant Breed Genet.

[CR4] Basu Majumder A, Bera B, Rajan A (2010). Tea statistics: global scenario. Inc J Tea Sci.

[CR5] Bhardwaj P, Kumar R, Sharma H, Tewari R, Ahuja PS, Sharma RK (2013). Development and utilization of genomic and genic microsatellite markers in Assam tea (*Camellia assamicassp*.*assamica*) and related *Camellia* species. Plant Breed.

[CR6] Brunings AM, Moyer C, Peres N, Folta KM (2010). Implementation of simple sequence repeat markers to genotype Florida strawberry varieties. Euphytica.

[CR7] Butler JM (2006). Genetics and genomics of core short tandem repeats loci used in human identity testing. J Forensic Sci.

[CR8] Butler JM, Crivellente F, McCord BR (2004). Forensic DNA typing by capillary electrophoresis: using the ABI Prism 310 and 3100 genetic analyzers for STR analysis. Electrophoresis.

[CR9] Chen L, Zhou ZX, Yang YJ (2007). Genetic improvement and breeding of tea plant (*Camellia sinensis*) in China: from individual selection to hybridization and molecular breeding. Euphytica.

[CR10] Cipriani G, Marrazzo MT, Di Gaspero G, Pfeiffer A, Morgante M, Testolin R (2008). A set of microsatellite markers with long core repeat optimized for grape *(Vitis* spp.) genotyping. BMC Plant Biol.

[CR11] Dettori MT, Micali S, Giovinazzi J, Scalabrin S, Verde I, Cipriani G (2015). Mining microsatellites in the peach genome: development of new long-core SSR markers for genetic analyses in five *Prunus* species. SpringerPlus.

[CR12] Divashuk MG, Klimushina MV, Karlov GI (2011). Molecular genetic characteristics of the Wx-B1e allele from common wheat and applicability of the DNA markers for its identification. Russ J Genet.

[CR13] Fang W, Cheng H, Duan Y, Jiang X, Li X (2012). Genetic diversity and relationship of clonal tea (*Camellia sinensis*) cultivars in China as revealed by SSR markers. Plant Syst Evol.

[CR14] Fang WP, Meinhardt LW, Tan HW, Zhou L, Mischke S, Zhang DP (2014). Varietal identification of tea (*Camellia sinensis*) using nanofluidic array of single nucleotide polymorphism (SNP) markers. Hortic Res.

[CR16] Gupta PK, Rustgi S, Kulwal PL (2005). Linkage disequilibrium and association studies in higher plants: present status and future prospects. Plant Mol Bio.

[CR17] Hasnaoui N, Buonamici A, Sebastiani F, Mars M, Zhang DP, Vendramin GG (2012). Molecular genetic diversity of *Punica granatum* L. (pomegranate) as revealed by microsatellite DNA markers (SSR). Gene.

[CR18] Hellmann AP, Rohleder U, Eichmann C, Pfeiffer I, Parson W, Schleenbecker U (2006). A proposal for standardization in forensic canine DNA typing: allele nomenclature of six canine-specific STR loci. J Forensic Sci.

[CR19] Hoda A, Vegara M, Bozgo V (2010). Genetic diversity of recka sheep breed in albania based on 15 microsatellite markers. Biotechnol Biotechnol Equip.

[CR20] Huo J, Yang G, Zhang Y, Li F (2013). A new strategy for identification of currant (*Ribes nigrum* L.) cultivars using RAPD markers. Genet Mol Res.

[CR21] Jian L, Zhang YZ, Yu DF, Zhu LQ (2010). Molecular characterization of *Cymbidium kanran* cultivars based on extended random amplified polymorphic DNA (ERAPD) markers. Afr J Biotechnol.

[CR22] Karaagac E, Yilma S, Cuesta-Marcos A, Vales MI (2014). Molecular analysis of potatoes from the Pacific Northwest tri-state variety development program and selection of markers for practical DNA fingerprinting applications. Am J Potato Res.

[CR23] Kaundun SS, Matsumoto S (2004). PCR-based amplicon length polymorphisms (ALPs) at microsatellite loci and indels from non-coding DNA regions of cloned genes as a means of authenticating commercial Japanese green teas. J Sci Food Agric.

[CR24] Li XY, Xu HX, Chen JW (2014). Rapid identification of red-flesh loquat cultivars using EST-SSR markers based on manual cultivar identification diagram strategy. Genet Mol Res.

[CR25] Liu K, Muse SV (2005). PowerMarker: an integrated analysis environment for genetic marker analysis. Bioinformatics.

[CR26] Liu GS, Zhang YG, Tao R, Fang JG, Dai HY (2014). Identification of apple cultivars on the basis of simple sequence repeat markers. Genet Mol Res.

[CR27] Ma JQ, Yao MZ, Ma CL, Wang XC, Jin JQ, Wang XM, Chen L (2014). Construction of a SSR-based genetic map and identification of QTLs for catechins content in tea plant (*Camellia sinensis*). PLoS One.

[CR28] Ma JQ, Huang L, Ma CL, Jin JQ, Li CF, Wang R Ka, Zheng HK, Yao MZ, Chen L (2015). Large-scale SNP discovery and genotyping or constructing a high-density genetic map of tea plant using specific-locus amplified ragment sequencing (SLAF-seq). PLoS One.

[CR29] Mejia EG, Ramirez-Mares MV, Puangpraphant S (2009). Bioactive components of tea: cancer, inflammation and behavior. Brain Behav Immun.

[CR30] Nei M, Tajima FA, Tateno Y (1983). Accuracy of estimated phylogenetic trees from molecular data. J Mol Evol.

[CR31] Pan L, Xia Q, Quan Z, Liu H, Ke W, Ding Y (2010). Development of novel EST–SSRs from sacred lotus (*Nelumbo nucifera Gaertn*) and their utilization for the genetic diversity analysis of *N. nucifera*. J Hered.

[CR32] Patzak J, Vrba L, Matousek J (2007). New STS molecular markers for assessment of genetic diversity and DNA fingerprinting in hop (*Humulus lupulus* L.). Genome.

[CR33] Reitz MS, Abrell JW, Trainor CD, Gallo RC (1972). Precipitation of nucleic acids with cetyltrimethylammonium bromide: a method for preparing viral and cellular DNA polymerase products for cesium sulfate density gradient analysis. Biochem Biophys Res Commun.

[CR34] Ruitberg CM, Reeder DJ, Butler JM (2001). STRBase: a short tandem repeat DNA database for the human identity testing community. Nucleic Acids Res.

[CR35] Sae-Lee N, Kerdchoechuen O, Laohakunjit N (2012). Chemical qualities and phenolic compounds of Assam tea after soil drench application of selenium and aluminium. Plant Soil.

[CR36] Sharangi AB (2009). Medicinal and therapeutic potentialities of tea (*Camellia sinensis* L.)—a review. Food Res Int.

[CR37] Taberlet P, Luikart G (1999). Non-invasive genetic sampling and individual identification. Biol J Linn Soc.

[CR38] Tamura K, Dudley J, Nei M, Kumar S (2007). MEGA4: molecular evolutionary genetics analysis (MEGA) software version 4.0. Mol Biol Evol.

[CR39] Tan LQ, Wang LY, Wei K, Zhang CC, Wu LY, Qi GN, Cheng H, Zhang Q, Cui QM, Liang JB (2013). Floral transcriptome sequencing for SSR marker development and linkage map construction in the tea plant (*Camellia sinensis*). PLoS One.

[CR40] Tan LQ, Peng M, Xu LY, Wang LY, Chen SX, Zou Y, Qi GN, Cheng H (2015). Fingerprinting 128 Chinese clonal tea cultivars using SSR markers provides new insights into their pedigree relationships. Tree Genet Genomes.

[CR41] Ujihara T, Ohta R, Hayashi N, Kohata K, Tanaka J (2009). Identification of Japanese and Chinese green tea cultivars by using simple sequence repeatmarkers to encourage proper labeling. Biosci Biotechnol Biochem.

[CR42] Verma P, Shah N, Bhatia S (2012). Development of an expressed gene catalogue and molecular markers from the de novo assembly of short sequence reads of the lentil (*Lens culinaris* Medik.) transcriptome. Plant Biotechnol J.

[CR43] Wachira FN, Waugh R, Powell W, Hackett CA (1995). Detection of genetic diversity in tea (*Camellia sinensis*) using RAPD markers. Genome.

[CR44] Wang XC, Zhao QY, Ma CL, Zhang ZH, Cao HL, Kong YM, Yue C, Hao XY, Chen L, Ma JQ, Jin JQ, Li X, Yang YJ (2013). Global transcriptome profiles of *Camellia sinensis* during cold acclimation. BMC Genom.

[CR45] Wu H, Chen D, Li J, Yu B, Qiao X, Huang H, He M (2012). De novo characterization of leaf transcriptome using 454 sequencing and development of EST-SSR markers in tea (*Camellia sinensis*). Plant Mol Biol Report.

[CR46] Yang XH, Xu YB, Shah T, Li HH, Han ZH, Li JS (2011). Comparison of SSRs and SNPs in assessment of genetic relatedness in maize. Genetica.

[CR47] Yang H, Wei CL, Liu HW, Wu JL, Li ZG, Zhang L, Jian JB, Li YY, Tai YL, Zhang J, Zhang ZZ, Jiang CJ, Xia T, Wan XC (2016). Genetic divergence between *Camellia sinensis* and Its wild relatives revealed via genome-wide SNPs from RAD sequencing. PLoS One.

[CR48] Yao MZ, Ma CL, Qiao TT, Jin JQ, Chen L (2011). Diversity distribution and population structure of tea germplasms in China revealed by EST-SSR markers. Tree Genet Genomes.

